# Experimental taphonomy of *Artemia* reveals the role of endogenous microbes in mediating decay and fossilization

**DOI:** 10.1098/rspb.2015.0476

**Published:** 2015-06-07

**Authors:** Aodhán D. Butler, John A. Cunningham, Graham E. Budd, Philip C. J. Donoghue

**Affiliations:** 1 School of Earth Sciences, University of Bristol, Life Sciences Building, 24 Tyndall Avenue, Bristol BS8 1TQ, UK; 2 Department of Earth Sciences, Palaeobiology Programme, Uppsala University, Villavägen 16, 75236 Uppsala, Sweden; 3 Department of Palaeobiology and Nordic Centre for Earth Evolution, Swedish Museum of Natural History, 10405 Stockholm, Sweden

**Keywords:** Cambrian explosion, palaeobiology, taphonomy, bilateria, metazoa

## Abstract

Exceptionally preserved fossils provide major insights into the evolutionary history of life. Microbial activity is thought to play a pivotal role in both the decay of organisms and the preservation of soft tissue in the fossil record, though this has been the subject of very little experimental investigation. To remedy this, we undertook an experimental study of the decay of the brine shrimp *Artemia*, examining the roles of autolysis, microbial activity, oxygen diffusion and reducing conditions. Our findings indicate that endogenous gut bacteria are the main factor controlling decay. Following gut wall rupture, but prior to cuticle failure, gut-derived microbes spread into the body cavity, consuming tissues and forming biofilms capable of mediating authigenic mineralization, that pseudomorph tissues and structures such as limbs and the haemocoel. These observations explain patterns observed in exceptionally preserved fossil arthropods. For example, guts are preserved relatively frequently, while preservation of other internal anatomy is rare. They also suggest that gut-derived microbes play a key role in the preservation of internal anatomy and that differential preservation between exceptional deposits might be because of factors that control autolysis and microbial activity. The findings also suggest that the evolution of a through gut and its bacterial microflora increased the potential for exceptional fossil preservation in bilaterians, providing one explanation for the extreme rarity of internal preservation in those animals that lack a through gut.

## 1. Introduction

The preservation of the soft-bodied anatomy of organisms in the fossil record occurs only rarely in *Konservat-Lagerstätten*. While such sites of exceptional preservation may be uncommon, their contributions to our knowledge of the history of life are disproportionate to their frequency [1]. It is ironic, therefore, that fossils exhibiting soft tissue preservation can be among the most difficult to interpret [2]. This is because fossils preserving soft tissues are invariably a melange of pristine to decayed structures preserved through mineral replication, while other aspects of anatomy are not preserved at all. Hence, the peculiarities of fossilization processes can lead to the preservation of different suites of anatomical structures, systematically distorting phylogenetic interpretations [3] but, by the same token, knowledge of such processes provides a framework for the correct interpretation of fossil remains.

It is generally accepted that the principal vectors of decay are autolysis and microbial activity, and that microbial activity is a key mediator of authigenic mineralization of soft tissue in the processes of exceptional fossilization [4]. However, the role of microbes has been treated largely as a black box that is not known or understood in detail, and the role of autolysis is essentially unknown in adult animals. Inspired by the discovery that animal embryos are rapidly pseudomorphed and stabilized by external bacterial biofilms that probably become the substrate for fossilization [5], and as bacteria are major mediators of exceptional soft tissue preservation [4,6], we set out to determine whether similar mechanisms might account for soft tissue preservation of complex and macroscopic bilaterian animals. Using the brine shrimp *Artemia salina* as our experimental organism, we analysed the pattern of autolytic and microbial decay, and biofilm development, under different environmental conditions that controlled for oxygen diffusion, exogenous microbial activity and/or fungal activity. Our results show that the carcass of the brine shrimp is consumed rapidly by gut-derived microbes after rupture of the gut wall and consequent release of endogenous bacteria to the rest of the body cavity, followed by microbially mediated tissue replacement through biofilm development, irrespective of external controls. These biofilms then mediate authigenic mineralization of soft tissues and of the biofilms themselves, within both the gut and body cavity. These findings suggest that internally derived microbes are the major control on internal preservation—a result that has important implications for our understanding of the fossil record of exceptionally preserved animals.

## 2. Material and methods

### (a) Justification of experimental organism

We selected the brine shrimp *Artemia salina* as the experimental organism, because its taphonomy has been well studied [7]; its translucent body allows microbial activity to be observed readily via optical microscopy; it is available cheaply and in large numbers, allowing statistically meaningful datasets to be obtained; and because arthropods dominate many *Konservat-Lagerstätten*, especially in Cambrian examples [8].

### (b) Experimental conditions

Animals were euthanized in standardized artificial seawater (ASW) taken from a marine aquarium with sediment and organisms which was first supersaturated with carbon dioxide and then sealed in an airtight Duran flask purged of remaining oxygen with a CO_2_/N_2_ gas mixture. Individual specimens were then transferred to 10 ml Wheaton serial crimp vials along with an aliquot of the ASW. Time-series replicates of five experimental systems were established in order to isolate the effects of autolysis and microbial activity on the decay and preservation of *Artemia*. The five experimental systems were set up as follows: (i) *open*—vials open to atmospheric diffusion, (ii) *closed*—sealed vials containing anoxic ASW, (iii) *reducing*—reducing environmental conditions consistent with an anoxic depositional environment with decay of organic matter by sulfate-reducing bacteria, simulated with 10 Mmol beta-mercaptoethanol, (iv) *antimicrobial*—10 µg ml^−1^ of rifampicin was introduced to anoxic ASW to control bacterial growth and (v) *reducing and antimicrobial*—inhibition of both exogenous microbial and autolytic processes by a combination of the conditions in (iii) and (iv). The antifungal reagent methylene blue was also used in the antimicrobial systems to prevent the opportunistic growth of fungi. The experiments were run for 35 days with samples taken at 1, 2, 3, 5, 7, 9, 12, 15, 25, 30 and 35 days. The systems were maintained at 15°C for the duration of the experiments.

### (c) Specimen preparation and analysis

For imaging, specimens were fixed in a solution of 2.5% gluteraldehyde and 2% formaldehyde in a 0.1 M sodium acetate buffer. A Leica binocular dissecting microscope, a Zeiss Photomicroscope III and a Leica M205C were used for light microscopy. Some specimens were stained with the fluorescent dye acridine orange. In fixed specimens, the emission spectrum of tissues shifts to red/orange, while bacteria appear green, allowing the two to be distinguished. Scanning electron microscope (SEM) analyses were carried out using a Hitachi S-3500 SEM at 15 keV, and decay experiment specimens were cryo-sectioned. Elemental data were collected using an EDAX *x*-ray dispersive (EDX) analysis system with Genesis software.

### (d) Semi-quantitative analyses

We captured the pattern of decay in a series of semi-quantitative indices [9] that delimit arbitrary points along the continuum of decay from complete organism to amorphous organic matter and allow us to describe the patterns of microbial and autolytic processes observed in gross morphology, internal tissue structure, internal microbial infiltration and surface microbial cover. Six specimens were removed from each of the five experimental systems and coded at each sampling interval. The use of these indices depends on description and assignment of a numerical value to each stage of decay (table 1).

**Table 1.** Description of semi-quantitative indices of decay.

gross morphology		internal anatomy	internal microbial infiltration index	surface microbial cover index
1	fresh (figure 1*a*)	fresh	no microbes in body cavity, restricted to gut (figure 2*a*)	none/isolated microbes
2	distal thoracopods shrivel and mat (figure 1*b*)	shrinkage of cells away from distal thoracopod setae, cells/nuclei in integument still visible under fluorescent microscopy, some evidence of degradation by autolysis	microbes escape from hindgut, microbes visible in haemocoel and telson (figure 2*b*)	isolated clusters of microbes visible
3	body appears opaque due to microbial growth inside the cuticle (figure 1*c*)	cellular details and outline degrade in integument, Z-banding no longer visible in muscle when viewed under cross-polarized light. Small droplets of what appear to be lipid form in association with tissues	microbes visible in distal appendages. Increase in density in haemocoel	patchy growth of biofilms (figure 2*g*)
4	shrinkage of cuticle and internal structure, loss of some distal appendages (figure 1*d*)	no cellular details visible, gross outline of muscle tissue still visible. Lipid droplets are present in large numbers in some cases outlining where tissue previously occurred	extensive infiltration by microbes into body cavity and through tissues. Individual microbes difficult to image (figure 2*c*)	enclosure—surface detail obscured by biofilm
5	thoracic and abdominal cuticle failure, internal contents leaks from carcass when disturbed (figure 1*e*)	no tissue structure visible except unsupported basement membrane of gut, large spherulitic masses of lipid proteinacous fragments visible	cuticle packed with biofilm, individual microbes impossible to image by light microscopy in appendages and body segments due to high population density (figure 5*a*)	thick biofilm of diverse morphotypes overgrows initial community, degradation of underlying cuticle surface evident (figure 2*h*)
6	cuticular fragments and unsupported gut all that remains (figure 1*f*–*g*)	structural failure, unsupported gut pulls away from cuticle, amorphous decay fabric released into surrounding medium	cuticle fails releasing microbes	structural failure of underlying cuticle

## 3. Results

### (a) Gross morphological decay

**Figure 1.**
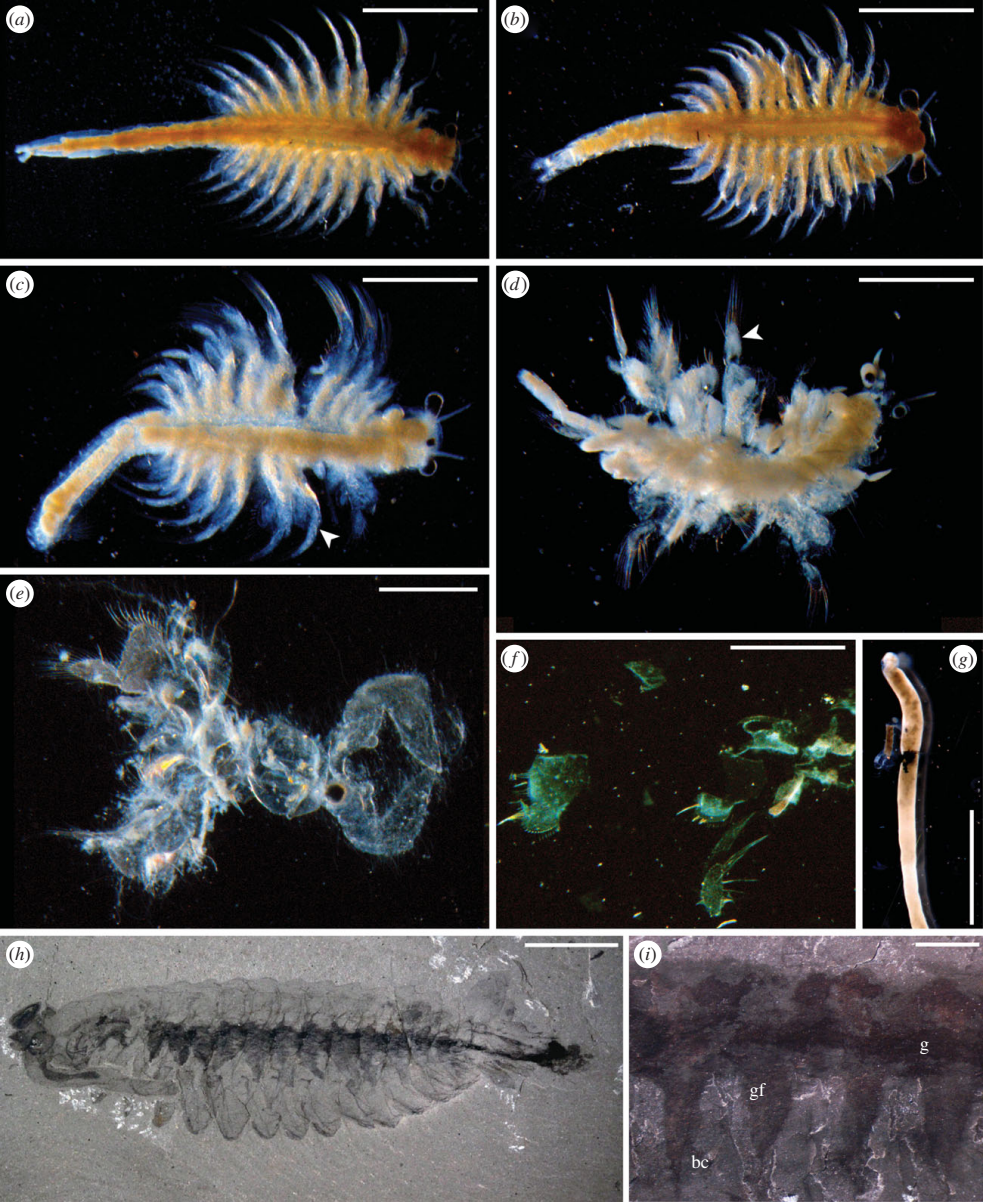
Generalized decay sequence in *Artemia* (*a*–*g*) and comparison to Burgess Shale-type fossils (*h*–*i*). (*a*) Undecayed specimen. (*b*) Thoracopods become matted. (*c*) Body and limbs (arrow) becomes opaque due to microbial activity. (*d*) Cuticle shrinks; some distal podomeres are disarticulated, cloudy appearance of limbs indicates internal biofilm. (*e*) Cuticle fails, internal biofilm is lost. (*f*) Cuticle disintegrates into fragments. (*g*) Only the unsupported gut remains. (*h*) *Opabinia* specimen (Burgess Shale, BC, Smithsonian National Museum of Natural History Washington DC, USNM 155600) showing the gut and associated microbial fabrics. (*i*) Enlargement of *Opabinia* gut area (Burgess Shale, BC, Geological Survey of Canada GSC 40251). Dark area, g gut; gf grey features corresponding to structures interpreted as gut diverticulae or limbs, bc lighter areas, body cavity. Scale bar: (*a*–*c*,*i*) = 2 mm, (*d*–*g*) = 1 mm, (*h*) = 10 mm. (Online version in colour.)

The overall pattern of morphological decay was not altered by the experimental treatments; only the rate at which decay progressed differed. The sequence of gross morphological decay conformed to the pattern described previously [7]. Initial signs of decay were observed in the distal portions of the thoracopods, which became shrivelled and matted (figure 1*b*). Next, the body became opaque because of microbial growth inside the cuticle (figure 1*c*). The cuticle began to shrink and the distal parts of some appendages disarticulated (figure 1*d*). Eventually, the thoracic and abdominal cuticle failed and contents leaked from the carcass (figure 1*e*). Decay proceeded until only cuticular fragments and an unsupported gut remained (figure 1*f*–*g*). Samples in open conditions decayed fastest, followed by closed, then reducing environments (figure 4*a*). The combination of reducing and antimicrobial treatments progressed at the slowest rate.

### (b) Tissue decay

**Figure 2.**
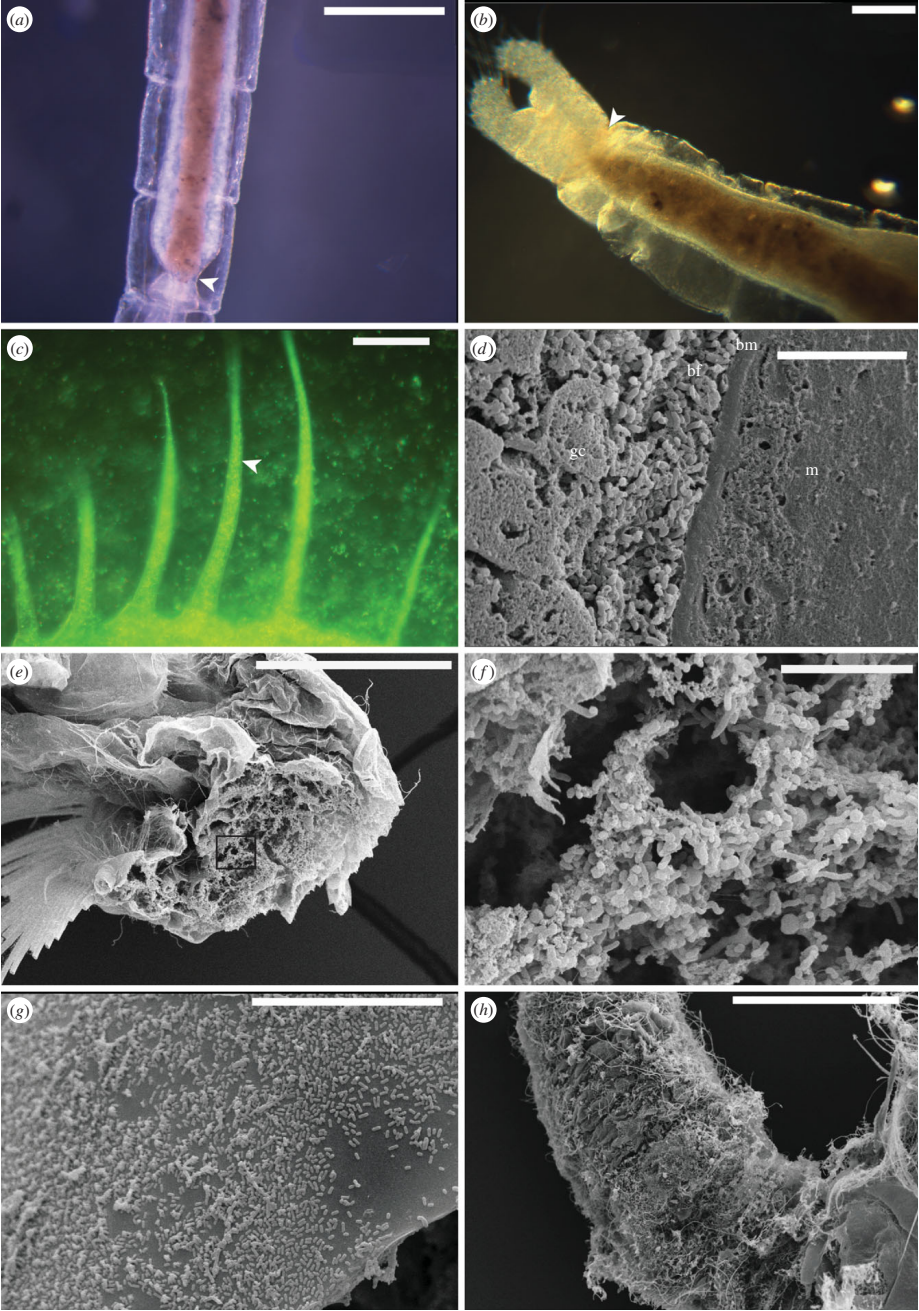
Microbial activity during *Artemia* decay. (*a*) Hindgut of live *Artemia*. (*b*) Microbes (arrow) escape at midgut/hindgut boundary and migrate into haemocoel. (*c*) Cuticle filled with microbes forming a dense biofilm; (*d*) replacement of gut epithelium by microbial pseudomorph. bm, basement membrane; bf, biofilm; gc, gut contents; m, musculature surrounding gut. (*e*) Section of appendage filled with biofilm (reducing conditions). (*f*) Enlargement of (*e*). (*g*) Surface microbes at early stage decay. (*h*) Diverse microbial community after 28 days in closed conditions. Scale bar (*a*) 500 µm, (*b*) 250 µm, (*c*) 50 µm, (*d*) 20 µm, (*e*) 200 µm, (*f*) 10 µm, (*g*) 20 µm, (*h*) 300 µm. (Online version in colour.)

Tissue decay occurred very rapidly in open conditions. The rate of decay decreased in a series from closed conditions, through reducing conditions, to reducing conditions with antimicrobial control. Anoxia alone was not sufficient to retard the decay of tissue by autolysis. It was only under reducing conditions, where autolytic tissue decay was greatly retarded, that tissues remained intact sufficiently long to become pseudomorphed by biofilm bacteria (figure 2*e*–*f*).

### (c) Microbial decay

Initially, and in all the systems, the bulk of microbial activity was restricted to the gut (figure 2*a*). Bacterial populations replaced the void left by decayed gut epithelial tissue in a distinct biofilm (figure 2*d*). Bacteria were prevented from further infiltration of the body cavity by the basement membrane underlying the gut. In all instances, failure of the epithelium occurred first at the interface of mid and hindgut regions (a possible weak point), where microbes were observed escaping into the body cavity (figure 2*b*). Here, they proliferated until the haemocoel was filled with a dense biofilm. Eventually, the entire body was filled by biofilm as internal tissues were replaced by microbes (figure 2*c,e,f*).

**Figure 3.**
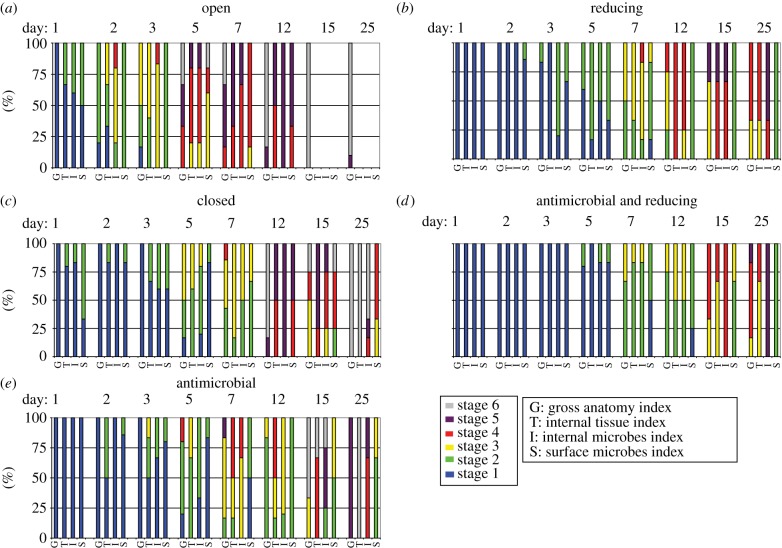
Count data of decay experiments, six samples coded per interval. (Online version in colour.)

**Figure 4.**
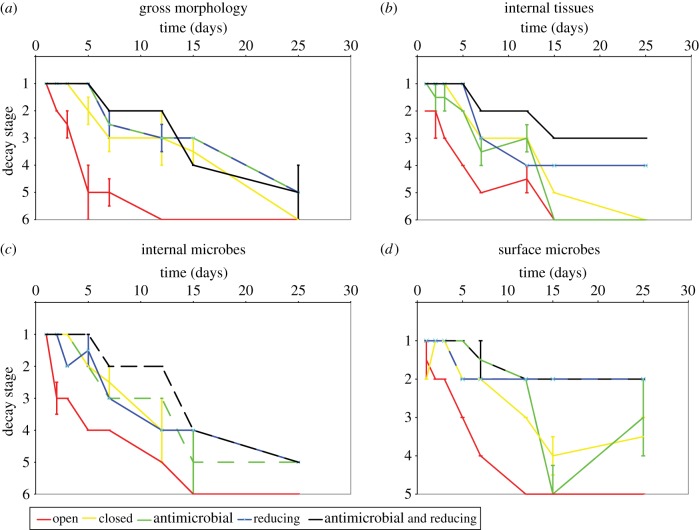
Graphs of median decay stage at each recorded time interval, error bars indicate median absolute deviation per interval where value is greater than zero. (Online version in colour.)

Externally, surface biofilms formed rapidly (within 1–2 days; figure 3), consisting initially of a relatively low diversity of morphotypes (figure 2*g*) that gradually engulfed the carcass with a diverse community of filamentous, rod and coccoid microbial morphotypes (figure 2*h*). Despite engulfing the carcass, the bacteria did not penetrate the interior until the cuticle failed, which invariably occurred after the body cavity had already been filled with endogenous gut-derived microbes (figure 3). Neither antimicrobial nor reducing conditions on their own resulted in a significant reduction in the rate of internal microbial infiltration of the gut bacteria (figure 4*b*). When reducing conditions were applied in tandem with antimicrobial controls the systems show a plateau that was possibly representative of a growth hiatus between seven and 12 days (figure 3). However, we failed to observe a difference between the systems with antimicrobial controls and the reducing and closed environments by the end of the experiment for this index.

Surface biofilm communities (figure 4*d*) were associated with a visible degradation of the cuticle in the later stages of decay (figure 2*h*). While their development was retarded in closed and antimicrobial conditions, the most effective inhibition of these surface microbial communities occurred in the reducing environmental conditions (figure 3), although they were still present (figure 2*e*). Inhibition in the activity of surface microbial communities in reducing conditions led to a reduced rate of gross morphological decay (figure 4*a*) of specimens compared with those that displayed extensive surface microbe colonization in the open (figure 4*d*) and closed systems.

### (d) Microbial biofilms and co-associated mineralization

**Figure 5.**
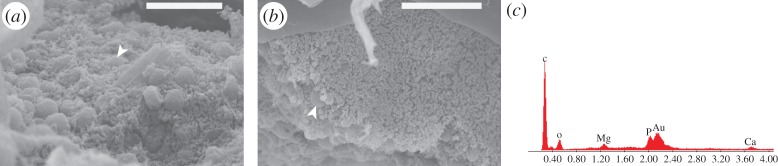
Biofilm associated with gut and internal body cavity with phosphatic mineral spheroids growing *in situ.* (*a*) Internal body cavity biofilm. (*b*) Gut-associated biofilm. Arrows indicate phosphatic spheroids. (*c*) Point EDX spectrum from mineralized spheroid, note distinct Ca, Mg and P peaks (not present in surrounding tissue) Au peak results from gold coating used for SEM imaging. Scale bar: (*a*) 20 µm and (*b*) 30 µm. (Online version in colour.)

A crystalline fabric was observed under cross-polarized light and SEM in association with the gut-derived biofilms in both the body cavity and gut itself (figures 2*d* and 5*a,b*). SEM-EDAX revealed enrichment of Ca, Mg and P in crystalline masses (figure 5*c*) suggesting early authigenic mineral formation of apatite or whitlockite in these biofilms.

## 4. Discussion

### (a) The role of autolysis, internal and external microbial activity in decay

Our experimental conditions were designed in order to explore the various influences of autolysis, endogenous and exogenous bacteria in the post-mortem degradation and biofilm replication of adult animal tissue level anatomy, providing important insights into the processes responsible for exceptional preservation. In the open conditions, both microbial and autolytic processes should proceed in an uninhibited way, and, indeed, this is reflected in our results. The lowest rate of tissue decay was observed under the reducing conditions, which have been shown previously to inhibit autolysis [10]. The stability of tissue structure for an extended period post-mortem was also associated with the replication of tissue structure by microbial pseudomorphs (figure 2*e*–*f*). In environmental conditions where autolysis was not inhibited, only homogeneous microbial fabrics were produced in the body cavity. This corroborates the previous suggestion that blocking autolysis is probably a necessary first step in the preservation of labile tissues [5,10].

In all experimental conditions, the wall of the gut failed at the mid- to hindgut junction, allowing gut-derived microbes to spread into the body where they proliferated. Under reducing conditions, where internal soft tissues remained intact because autolytic degradation was blocked, the gut-derived microbes replicated these tissues. This implies a key role for gut bacteria in the stabilization of tissue and organ level anatomy, as part of the process of exceptional preservation through mineral replication. Exogenous microbes always played a subordinate role, regardless of the experimental conditions. Microbes derived from the gut had already filled the body by the time the cuticle failed allowing exogenous microbes to enter. We propose, therefore, that gut-derived microbes are key to the replication of internal anatomy. However, because of the importance of the cuticle in retaining structural integrity of the decaying organism, limiting the activity of surface microbial activity that degrades cuticle also improves the preservation potential of a cuticular organism. Size differences and variations in cuticle thickness probably also affect preservation potential between separate taxa, but since a similar pattern of gut rupture and similar progression of decay sequence is also observed in decay experiments of velvet worms [11], the primacy of gut-derived microbial decay is probably universal, at least among ecdysozoans.

### (b) The role of microbes in internal preservation

The fact that there is evidence for mineralization associated with the biofilms leads us to suggest that internal biofilms are the precursors to exceptional preservation through the mineral replication of soft tissue anatomy. This notion is supported by previous studies showing that microbial fabrics of the kind observed in our experiments can produce high-fidelity pseudomorphs of soft tissues that are robust (at least on a small scale) [5,12,13] and are able to mediate mineralization [5,6]. Microbially mediated mineralization can probably occur in the absence of pseudomorphing, resulting in the ‘substrate microfabrics’ that lack direct evidence of bacteria [14].

**Figure 6.**
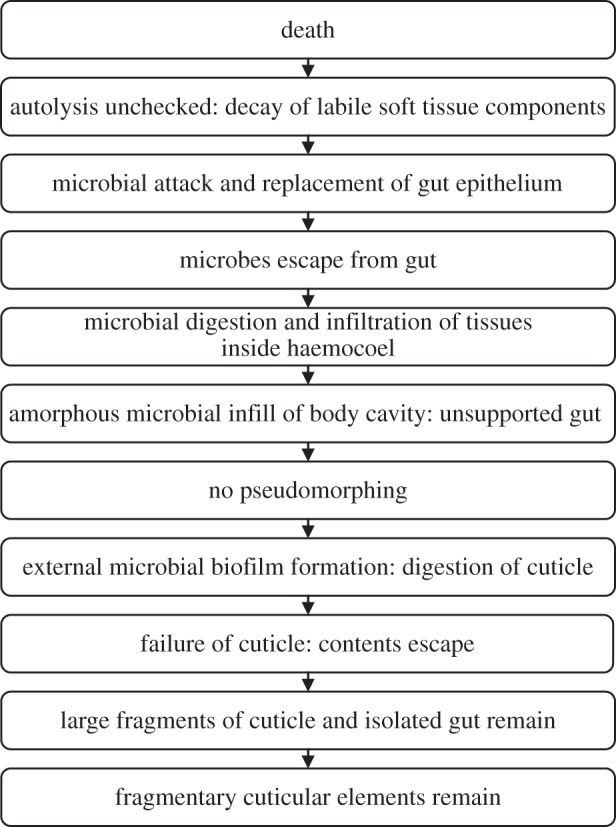
General model of decay in *Artemia* in open conditions.

Unlike experiments on animal embryos [5,13], where the biofilm-forming bacteria must be externally derived, in *Artemia* the endogenous gut bacteria are sufficient to control decay, stabilization and, ultimately, the preservation of soft tissues. Autolysis of tissues prior to stabilization by pseudomorphing or microbially mediated mineralization may explain why the gut microenvironment is often preserved in three dimensions in deposits of exceptional preservation, whereas tissues associated with the body cavity are often much more poorly preserved, if at all [15]. Although the gut, especially if it is infilled at the time of death, may be readily stabilized by the presence of bacteria from the start of decay, the ability of the gut-derived bacteria to capture other features of internal anatomy may depend on the extent of autolysis of non-gut tissues at the time of gut rupture. If the rupture of the gut is delayed then, clearly, there may be little remaining of the original biological structure to serve as a substrate for mineral replication, save amorphous remains (figure 6). For example, in some Sirius Passet and Burgess Shale arthropods (figure 1*h*–*i*) the gut is preserved and the morphology of internal structures is lost, with only amorphous features remaining. These may have been interpreted previously as limb extensions [16,17] or gut diverticulae [15,18] but based on our experimental observations and the resemblance to decay derived fabrics (figure 1), these structures may represent remnants of highly decayed tissues and their associated microbial biofilm-derived fabrics.

Fossilized internal biofilms have been observed directly in animal remains from throughout the Phanerozoic, including hyolith guts [19] and also *Mesolimulus*, an exceptionally preserved arthropod from the Upper Jurassic of Germany [20], where biofilms fill the haemocoel enveloping authigenically mineralized muscles, as in our experimental results. Fossilized guts are relatively common in *Konservat-Lagerstätten* with observations from *Ottoia*, for example, showing that the gut is retained in 75% of individuals [21]. Indeed, guts are regularly the only portions of the internal anatomy that are preserved in fossils from these localities [21–24]. We recognize that the gut itself is readily fossilized as a product of both its unique microenvironmental conditions, including high phosphate content and endogenous microbial flora. Our results provide experimental support that this is the fossilization mechanism for bilaterian guts. The preservation of detailed internal anatomy is much rarer, although records of such preservation do exist, for example, musculature from annelids [25] and arthropods [26] as well as nervous tissue [27].

The patterns observed in the fossil record are, therefore, compatible with those identified in our experiments. Bacteria colonize the gut in living organisms and rapidly form biofilms in the gut post-mortem, which is preserved comparatively frequently. On the other hand, biofilms can only pseudomorph other features if the conditions allow them to do so before the structures decay. Hence, apart from the gut, internal anatomy is rarely preserved [7]. Given this role for endogenous gut bacteria in mediating exceptional preservation, we propose that an endogenous source of bacteria may be essential for preservation of internal anatomy, as seen in *Konservat-Lagerstätten*. The distribution of ‘substrate’ and ‘microbial’ preservation fabrics [14] shows a degree of taxonomic control, stemming from differential rates of microbial penetration of carcasses. We hypothesize that this taxonomic control is the result of the intrinsic arrangements of a body plan altering the trajectory of microbial infiltration, e.g. open versus closed circulatory systems, an open haemocoel providing an ideal environment for microbes to proliferate, for example.

### (c) Reconciling differences between *Konservat-Lagerstätten*

**Figure 7.**
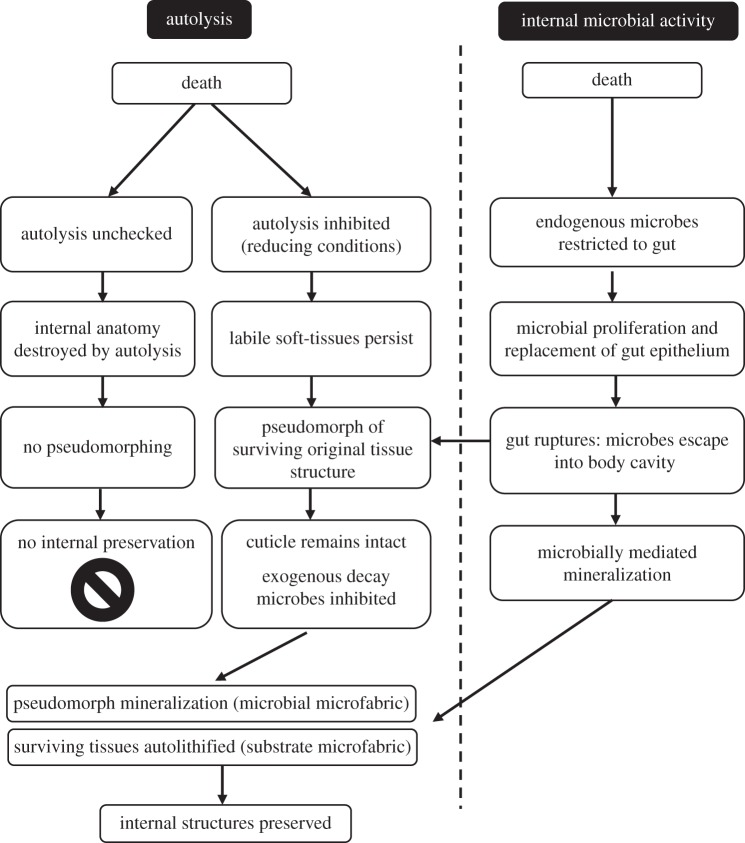
Hypothesized model of decay and pathways leading to exceptional preservation.

*Konservat-Lagerstätten* characterized by exceptional preservation are not generated equally. Instead, each fauna, indeed, each individual organism, can be viewed as a separate exit point from the normal progression of decay prior to fossilization. It is the extent of autolysis versus microbial activity and biofilm stabilization of anatomical structure that may account for the disparity of anatomical structures present in individual exceptionally preserved fossils. While the relative volatility and differential susceptibility of soft tissues to decay have been studied previously [4,28], the mechanisms underlying this are less apparent. We propose that our taphonomic model provides a deductive framework in which to understand the nature of taphonomic processes on soft tissue anatomy post-mortem and, consequently, inform the interpretation of exceptionally preserved fossils more generally. For example, in cases where highly labile structures are preserved by authigenic mineralization, where autolysis is inhibited and pseudomorphs that stabilize internal anatomy have the opportunity to form (figure 7). Conversely, in cases where autolysis continues unchecked in open environments, decay obliterates internal anatomy rapidly before stabilizing biofilms can form, leaving an unsupported gut in a sack of amorphous decayed tissue (figure 6). If decay progresses unchecked, eventually external biofilms and decay mediating organisms digest and degrade the cuticle, that then ruptures and fragments (figure 7). Thus, the preservational state of a given fossil reflects its trajectory exiting the more general decay pathway, from exceptionally preserved Burgess Shale arthropods e.g. *Branchiocaris* and *Opabinia* (figure 1*h*–*i*), through branchiopods and notostracans from the Upper Devonian Strud locality of Belgium [29] to the small carbonaceous fossil record of recalcitrant cuticular structures such as mandibles [30]. The finest level, and likely limit of fossilization, that of organic preservation of labile soft tissues, is restricted to highly specialized settings and confined to specific tissues, e.g. red bone marrow in frogs from Libros, Spain [31], reflecting stage 2 of internal tissue preservation index in our model. More common classic examples including Mesozoic Plattenkalk deposits, such as those from Solnhofen, Nüsplingen, Cerin, Lebanon or the Santana Formation, preserve internal muscle tissue relatively frequently [20,32–35]. Cambrian *Konservat-Lagerstätten* preserve muscle tissue only rarely in Sirius Passet arthropods and annelids [24,25]. These exhibit high-fidelity internal preservation through inhibition of autolysis and exogenous microbial decay, allowing endogenous biofilms to pseudomorph structures and catalyse authigenic mineralization, equivalent to stages 3–4 (figure 6). However, the effects of decay are readily apparent and information loss may preferentially act on characters that are important for taxonomic classification [3,4].

Further along the decay pathway, Orsten phosphacopids retain a cuticle, but internally preserve an unsupported gut and a spheroidal decay fabric [36], corresponding to stage 4, with muscles preserved rarely in low resolution (figure 6). The effects of autolysis and microbial decay may have progressed much further in both the Strud and Burgess Shale preservational settings, eliminating structural information such as musculature and most internal organs before fossilization occurred, leaving only recalcitrant gut and cuticle structures relatively intact with amorphous decay derived fabrics replacing the majority of soft tissues (internal decay stages 4–5). The extensive record of small carbonaceous fossils [30], i.e. that of disarticulated cuticle fragments, corresponds to the end stage in our model.

### (d) The role of a through gut in modifying preservation potential

The key role of gut-derived microbes in decay and, by inference, preservation means that the evolution of a through gut is likely to have important implications for preservation potential. Organisms that have blind guts, such as cnidarians, evert their guts such that they cannot maintain a gut flora. As a result, one might expect that such organisms would have little chance of preserving internal anatomy. Preservation must depend on the formation of favourable external biofilms that invade inwards, similar to the process observed in embryos, to stabilize their internal anatomical structure post-mortem, allowing a much longer window for internal autolytic processes to take effect and thus resulting in a much lower preservation potential for internal anatomy. This prediction is largely borne out by the fossil record. The overall quality of preservation is also often of a lower fidelity in described soft-bodied diploblast grade and blind-gut bearing organisms relative to groups possessing through-guts. For example, arthropods, annelids, priapulids and hyoliths can in many cases preserve aspects of gut, musculature and, in rare cases, neural tissues. On the other hand, diploblastic organisms, such as cnidarians, are typically found as impressions or outlines only (with the notable exception of very rare specimens of the probable cnidarian *Olivooides* [37,38]). This may go some way to explain the mismatch between phylogenetic and molecular clock expectations that diploblasts existed long before diploblast bilaterians, yet the fossil records of diploblast and triploblast eumetazoans is approximately coincident [39,40].

Under almost all circumstances, pseudomorphing of biological anatomy by biofilm-forming microbes [5,13] may be limited to small structures. This process can provide a good explanation for the preservation of microfossils such as fossilized embryos as well as internal microenvironments, such as guts, within larger fossils. However, it is only in the most exceptional examples of exceptional fossil preservation that microbes replicate and preserve internal anatomy more generally. Bacterial biofilm pseudomorphing of anatomical structure may not be an important mechanism in preserving macroscale animal remains, even though endogenous microbes are important vectors of the decay of visceral tissues that leaves cuticle articulated and intact in Burgess Shale-type preservation. Thus, endogenous microbes exert a fundamental control on the amount of soft tissue morphology, and therefore the amount of anatomical information, that is preserved in *Konservat-Lagerstätten*. Hence, the evolution of a through gut is an important factor in both the ecology of metazoan diversification and its fossil record. This finding also suggests the bauplan of an animal may act as a strong control on the processes of subsequent taphonomic transformation into an exceptionally preserved fossil, when the basic conditions required for the genesis of *Konservat-Lagerstätten* are met.

## Data accessibility

The datasets supporting this article are available at doi:10.5061/dryad.f0tt2.
